# MHD Casson Fluid Flow over a Stretching Sheet with Entropy Generation Analysis and Hall Influence

**DOI:** 10.3390/e21060592

**Published:** 2019-06-14

**Authors:** Mohamed Abd El-Aziz, Ahmed A. Afify

**Affiliations:** 1 Department of Mathematics, Faculty of Science, King Khalid University, Abha 9004, Saudi Arabia; 2 Department of Mathematics, Deanship of Educational Services, Qassim University, P.O. Box 6595, Buraidah 51452, Saudi Arabia

**Keywords:** MHD Casson fluid, slip factor, Hall current, entropy generation, Bejan number

## Abstract

The impacts of entropy generation and Hall current on MHD Casson fluid over a stretching surface with velocity slip factor have been numerically analyzed. Numerical work for the governing equations is established by using a shooting method with a fourth-order Runge–Kutta integration scheme. The outcomes show that the entropy generation is enhanced with a magnetic parameter, Reynolds number and group parameter. Further, the reverse behavior is observed with the Hall parameter, Eckert number, Casson parameter and slip factor. Also, it is viewed that Bejan number reduces with a group parameter.

## 1. Introduction

The study of magnetohydrodynamic flows with Hall currents has evinced interest attributable to its numerous applications in industries, such as MHD power generators, Hall current accelerators, Hall current sensors, and planetary fluid dynamics. Sato [1] was the first author who investigated the impact of Hall current on the flow of ionized gas between two parallel plates. The influence of Hall current on the efficiency of an MHD generator was investigated by Sherman and Sutton [2]. Several authors [3,4,5,6,7,8,9] discussed the influence of Hall current on hydromagnetic flow problems for various aspects. Recently, Abdel-Wahed [10] examined the impacts of hall current on the MHD boundary layer flow and heat transfer of Ferro nanofluid in a curved tube.

Some different fluids are termed as non-Newtonian fluids such as Jeffrey fluid, viscoelastic fluid, power-law flow, Williamson fluid, micropolar fluid, and Casson fluid. Casson [11] was the first investigator who introduced the Casson fluid model. Reviews of Casson fluid over different geometries have been presented in Refs. [12,13,14,15,16,17]. Recently, Ramana Reddy et al. [18] numerically analyzed the combined influences of thermal radiation and viscous dissipation of a paraboloid along an upper convective surface. All the above previous researchers have employed the first law of thermodynamics only. On the other hand, the second law of thermodynamics is utilized to minimize the entropy generation in thermal engineering systems. Entropy generation analysis in applied thermal engineering was proposed by Bejan [19,20,21]. Later on, many researchers [22,23,24,25,26,27,28,29,30,31] have effectively applied his approach to calculating the entropy generation analysis for different geometrical configurations. Recently, Reddy et al. [32] investigated the entropy generation for MHD Casson fluid flow with thermal radiation influence. Very recently, Afridi et al. [33] discussed the second law analysis for MHD flow and heat transfer past a slender stretching surface by taking Joule heating and variable thickness. To our knowledge, no document has yet been initiated for the MHD boundary layer of a Casson fluid owing to a stretching surface considering Hall effect, slip phenomenon and entropy generation. The objective of the present document is to discuss the second law of thermodynamics for a Casson fluid flow along a stretching surface taking the Hall current, velocity slip, and viscous dissipation influences. The impact of physical parameters is analyzed with the help of graphs and tables. 

## 2. Mathematical Formulation

**Figure 1:**
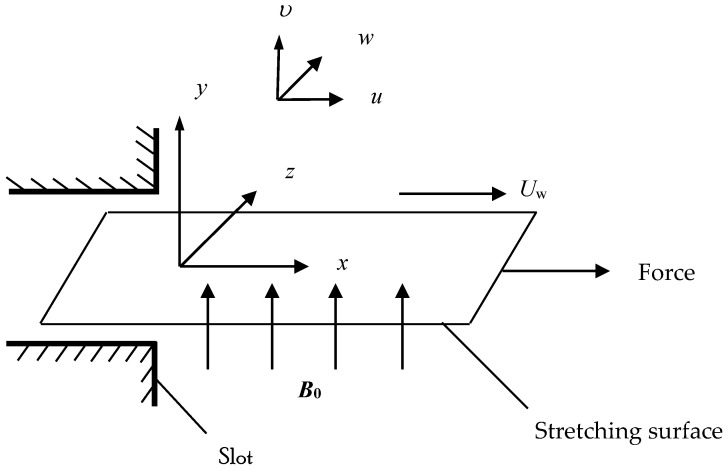
Physical model and coordinate system.

In this paper, the magnetohydrodynamic flow of incompressible Casson fluid is considered. The flow is generated owing to the stretching surface with linear velocity $u_{w}(x)=cx$, in the x-direction. Hall current is produced due to the strong magnetic field which is vertical to the stretching surface in the y-direction, as shown in Figure 1. The induced magnetic field is ignored with respect to small magnetic Reynolds number. The heat transfer characteristic is examined via viscous dissipation. Bejan number and entropy generation are utilized to evaluate the loss of energy for the existing flow regime. Further, it is assumed that the Joule heating is neglected in this study. The generalized Ohm’s law, including Hall current, is stated in the form Sutton and Sherman [2]:

(1) $$\vec{J}+\frac{\omega_{e}\tau_{e}}{B_{0}}\left(\vec{J}\times \vec{B}\right)=\sigma \left(\vec{E}+\vec{V}\times \vec{B}\right)$$

where $\vec{J}=\left(J_{x},J_{y},J_{z}\right)$, $\vec{V}$, $\vec{E}$, $\vec{B}=\left(0,B_{0},0\right)$, $\tau_{e}$, $\omega_{e}$, $\sigma \left(=\frac{e^{2}n_{e}\tau_{e}}{m_{e}}\right)$, *e*, $n_{e}$ and $m_{e}$ are the current density vector, the velocity vector, the electric field vector, the magnetic induction vector, the electron collision time, the cyclotron frequency of electron, the electrical conductivity of the fluid, the charge of electron, the number density of electrons, and the mass of the electron, respectively. In this work, an electric field is neglected, thus Equation (1) becomes:

(2) $$J_{x}=\frac{\sigma B_{0}}{1+m^{2}}\left(mu-w\right)$$

(3) $$J_{z}=\frac{\sigma B_{0}}{1+m^{2}}\left(mw+u\right)$$

where, $m=\omega_{e}\tau_{e}$ is the Hall parameter. 

According to Refs [12,13], the rheological equation of the Casson fluid is given by:

(4) $$\tau_{ij}=\left\{ \begin{array}{l} 2\left(\mu_{B}+\frac{p_{y}}{\sqrt{2\pi }}\right)e_{ij},\;\pi \;>\;\pi_{c}\\ 2\left(\mu_{B}+\frac{p_{y}}{\sqrt{2\pi_{c}}}\right)e_{ij},\pi \;<\;\pi_{c} \end{array}\right.$$

where $\pi =e_{ij}e_{ij}$ with $e_{ij}$ being the (i, j)^th^ component of the deformation rate, π depicts the product of the component of the deformation rate with itself, $\pi_{c}$ denotes a critical value of this product based on the non-Newtonian model, $\mu_{B}$ indicates the plastic dynamic viscosity of non-Newtonian fluids, and $p_{y}$ is the yield stress of the fluid. When $\pi <\pi_{c}$, Equation (4) can be expressed as: $\tau_{ij}=\mu_{B}\left(1+\frac{1}{\gamma }\right)\left(2e_{ij}\right)$. Here $\gamma =\frac{\mu_{B}\sqrt{2\pi_{c}}}{p_{y}}$ is the Casson parameter. 

Due to the above-mentioned assumptions and the boundary layer approximations, the governing equations of Casson fluid and generalized Ohm’s law with Hall current influence are given by:

(5) $$\frac{\partial u}{\partial x}+\frac{\partial v}{\partial y}=0$$

(6) $$u\frac{\partial u}{\partial x}+v\frac{\partial u}{\partial y}=\upsilon \left(1+\frac{1}{\gamma }\right)\frac{\partial^{2}u}{\partial y^{2}}-\frac{\sigma B_{0}^{2}}{\rho \left(1+m^{2}\right)}\left(u+mw\right)$$

(7) $$u\frac{\partial w}{\partial x}+v\frac{\partial w}{\partial y}=\upsilon \left(1+\frac{1}{\gamma }\right)\frac{\partial^{2}w}{\partial y^{2}}+\frac{\sigma B_{0}^{2}}{\rho \left(1+m^{2}\right)}\left(mu-w\right)$$

(8) $$u\frac{\partial T}{\partial x}+v\frac{\partial T}{\partial y}=\alpha \frac{\partial^{2}T}{\partial y^{2}}+\frac{\upsilon }{c_{p}}\left(1+\frac{1}{\gamma }\right)\left\{{\left(\frac{\partial u}{\partial y}\right)}^{2}+{\left(\frac{\partial w}{\partial y}\right)}^{2}\right\}$$

Subject to the boundary conditions:

(9) $$\begin{array}{l} u=u_{w}+\left(1+\frac{1}{\gamma }\right)L\frac{\partial u}{\partial y},\;v=0,\;w=\left(1+\frac{1}{\gamma }\right)L\frac{\partial w}{\partial y},\;T=T_{w}(x)=T_{\infty }+bx^{2}\;\mathrm{at}\;y=0,\;\\ u=w=0,\;T=T_{\infty }\;as\;y\to \infty \end{array}$$

The following non-dimensional variables are defined as:

(10) $$\eta ={\left(\frac{c}{\upsilon }\right)}^{1/2}y,\;u=c\;x\;f^{\prime }\left(\eta \right),\;v=-{\left(c\;\upsilon \right)}^{1/2}\;f\left(\eta \right),\;w=c\;x\;h\left(\eta \right),\theta \left(\eta \right)=\frac{T-T_{\infty }}{T_{w}-T_{\infty }}$$

where $\upsilon =\mu_{B}/\rho$, $\alpha =k/\rho c_{p}$, *k*, *ρ*, $c_{p}$, $T_{w}$, $T_{\infty }$, *b*, *c*, *L*, *f*, and *h,* are the kinematic viscosity, thermal diffusivity, thermal conductivity, fluid density, specific heat, temperature at the surface, ambient temperature, positive constants, characteristic length, dimensionless stream function, and dimensionless transverse velocity, respectively. By invoking Equation (10), Equation (5) is automatically satisfied whereas the other equations and the boundary condition take the following form:

(11) $$\left(1+\frac{1}{\gamma }\right)f^{\prime \prime \prime }+ff^{\prime \prime }-{f^{\prime }}^{2}-\frac{M}{1+m^{2}}\left(f^{\prime }+mh\right)=0$$

(12) $$\left(1+\frac{1}{\gamma }\right)h^{\prime \prime }+fh^{\prime }-f^{\prime }h+\frac{M}{1+m^{2}}\left(mf^{\prime }-h\right)=0$$

(13) $$\frac{1}{\mathrm{Pr}}\theta^{\prime \prime }+f\theta^{\prime }-2f^{\prime }\theta +Ec\left(1+\frac{1}{\gamma }\right)\left({f^{\prime \prime }}^{2}+{h^{\prime }}^{2}\right)=0$$

(14) $$\begin{array}{l} f\left(0\right)=0,\;f^{\prime }\left(0\right)=1+\chi \left(1+\frac{1}{\gamma }\right)f^{\prime \prime }(0),\;h\left(0\right)=\chi \left(1+\frac{1}{\gamma }\right)h^{\prime }(0),\;\theta \left(0\right)=1,\;\\ f^{\prime }\left(\infty \right)=\;0,\;h\left(\infty \right)=\;0,\;\theta \left(\infty \right)=\;0 \end{array}$$

Here, prime denotes differentiation with respect to η, f is a dimensionless stream function, h is the dimensionless transverse velocity, θ is the dimensionless temperature, $\mathrm{Pr}=\frac{\upsilon }{\alpha }$ is Prandtl number, $M=\frac{\sigma B_{0}^{2}}{\rho c}$ is a magnetic parameter, $\chi =L{\left(\frac{c}{\upsilon }\right)}^{1/2}$ is the slip parameter, $Ec=\frac{c^{2}}{bc_{p}}$ is the Eckert number, and m is the Hall parameter. The quantities of physical interest in this problem are the local skin friction coefficient in the *x*-direction $C_{fx}$, the local skin friction coefficient in the *z*-direction $C_{fz}$ and the local Nusselt number $Nu_{x}$ which are defined as:

(15) $$C_{fx}=\frac{\tau_{wx}}{\rho u_{w}^{2}},C_{fz}=\frac{\tau_{wz}}{\rho u_{w}^{2}},Nu_{x}=\frac{xq_{w}}{k\left(T_{w}-T_{\infty }\right)}$$

where $\tau_{wx}$ and $\tau_{wz}$ are the surface shear stresses in the *x*-and *z*-directions, respectively, and $q_{w}$ is the surface heat flux which is given by following the relations:

(16) $$\tau_{wx}=\left(\mu_{B}+\frac{p_{y}}{\sqrt{2\pi_{c}}}\right){\left(\frac{\partial u}{\partial y}\right)}_{y=0},\tau_{wz}=\left(\mu_{B}+\frac{p_{y}}{\sqrt{2\pi_{c}}}\right){\left(\frac{\partial w}{\partial y}\right)}_{y=0},q_{w}=-k{\left(\frac{\partial T}{\partial y}\right)}_{y=0}$$

Using the similarity and dimensionless variables (10), we get:

(17) Rex1/2Cfx=(1+1γ)f″(0),Rex1/2Cfz=(1+1γ)h′(0),NuxRex1/2=−θ′(0)

where, Rex=x uwυ is the local Reynolds number.

## 3. Entropy Generation Analysis

The local entropy generation rate is defined as Bejan ([20,21]):

(18) $$S_{gen}^{\prime \prime \prime }=\frac{k}{T_{\infty }^{2}}{\left(\frac{\partial T}{\partial y}\right)}^{2}+\frac{\mu_{B}}{T_{\infty }}\left(1+\frac{1}{\gamma }\right)\left[{\left(\frac{\partial u}{\partial y}\right)}^{2}+{\left(\frac{\partial w}{\partial y}\right)}^{2}\right]+\frac{\mu_{B}}{T_{\infty }}\frac{\sigma B_{0}^{2}}{\left(1+m^{2}\right)}\left(u^{2}+w^{2}\right)$$

In the entropy equation, the first term represents the heat transfer irreversibility, second term the fluid friction, and the last term due to the impact of the magnetic field. 

The characteristic entropy generation rate is expressed as:

(19) $$S_{0}^{\prime \prime \prime }=\frac{k{\left(\Delta T\right)}^{2}}{L^{2}T_{\infty }^{2}}$$

The dimensionless entropy generation can be expressed as follows:

(20) NG=Sgen″′S0″′=ReLθ′2(η)+MReL(1+m2)(BrΩ)(f′2(η)+h2(η))+ReL(1+1γ)(BrΩ)(f″2(η)+h′2(η))

where, ReL=c L2υ is the Reynolds number, $Br=\frac{\mu_{B}\;u_{w}^{2}}{k\Delta T}$ is the Brinkman number, $\Omega =\frac{\Delta T}{T_{\infty }}$ is the dimensionless temperature difference parameter, and $\Delta T=\left(T_{w}-T_{\infty }\right)$ is the temperature difference. Equation (20) can be expressed as:

(21) $$N_{G}=N_{1}+N_{2}$$

where N1=ReLθ′2(η) and N2=MReL(1+m2)(BrΩ)(f′2(η)+h2(η))+ReL(1+1γ)(BrΩ)(f″2(η)+h′2(η)) indicate the irreversibility due to heat transfer and the entropy generation due to the fluid friction with the magnetic field, respectively. Bejan number is introduced as:

(22) $$Be=\frac{N_{1}}{N_{G}}=\frac{1}{1+\Phi }$$

From Equation (22), Bejan number is in the range 0≤Be≤1. Therefore, 0 ≤ Φ ≤ 1 shows that the irreversibility is primarily owing to the heat transfer irreversibility, whereas for Φ > 1 it is owing to the fluid friction irreversibility. 

## 4. Results and Discussions 

**Figure 2:**
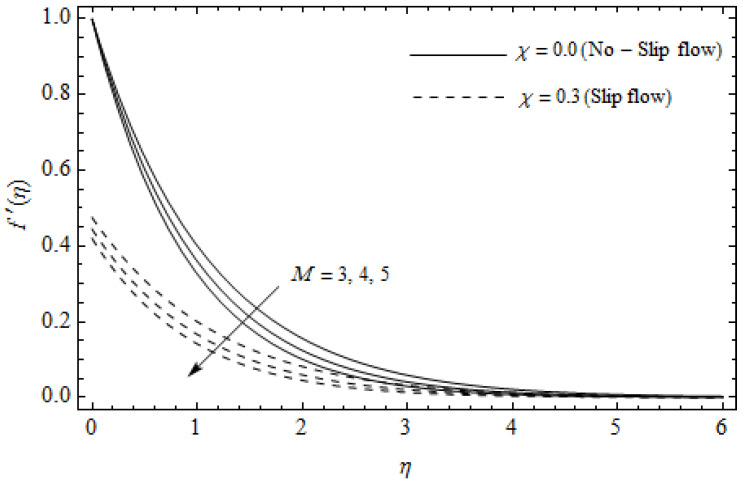
Axial velocity profiles for different values of magnetic parameter *M* and slip factor χ with γ = 0.3, *m*=0.5, *Ec*=0.0 and *Pr* = 2.

**Figure 3:**
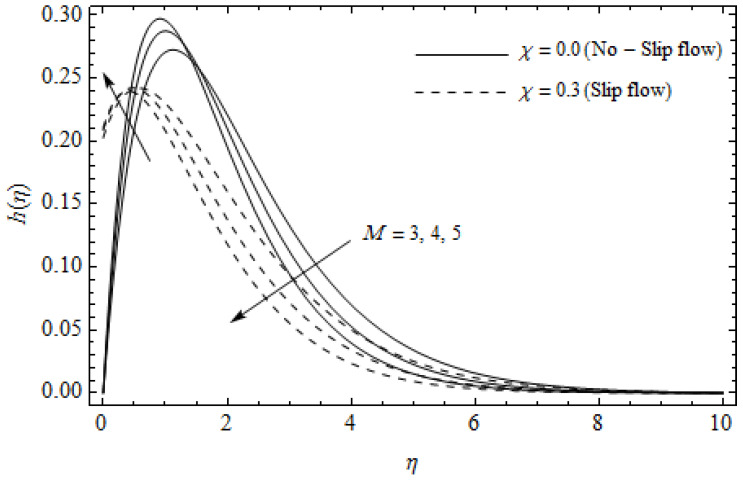
Secondary velocity profiles for different values of magnetic parameter *M* and slip factor χ with γ = 0.3, *m* = 0.5, *Ec* = 0.0 and *Pr* = 2.

**Figure 4:**
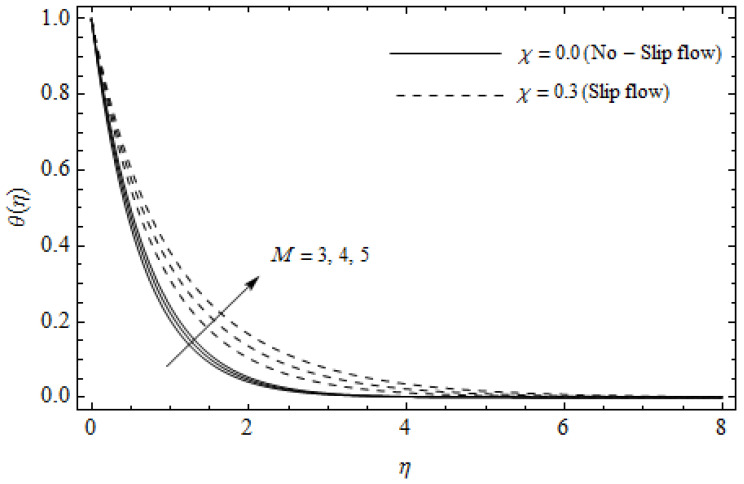
Temperature profiles for different values of magnetic parameter *M* and slip factor χ with γ = 0.3, m=0.5, Ec=0.0 and *Pr* = 2.

**Figure 5:**
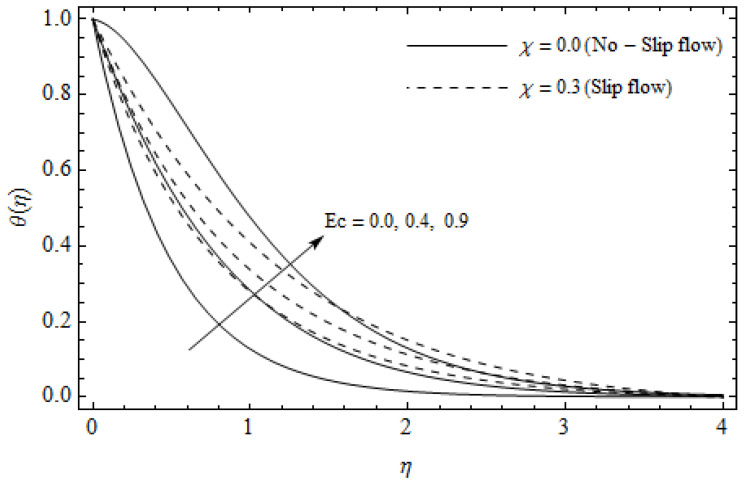
Temperature profiles for different values of Eckert number Ec and slip parameter χ with γ = 0.3, *m* = 0.5, M=3 and *Pr* =2.

**Figure 6:**
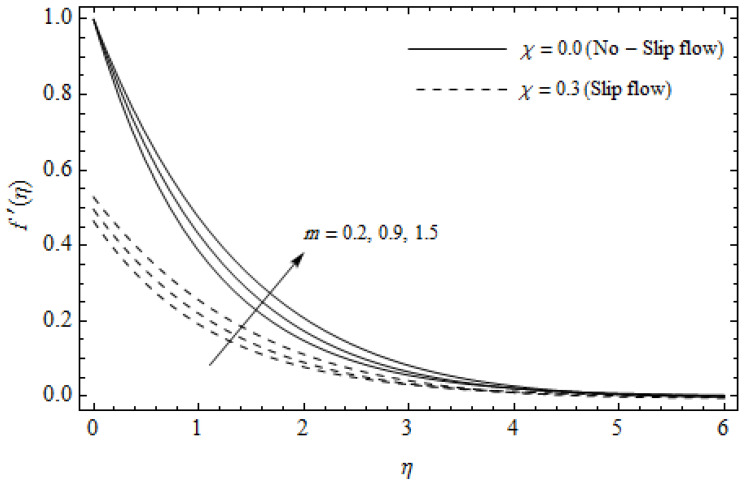
Axial velocity profiles for different values of Hall parameter *m* and slip parameter χ with γ = 0.3, M=3, *m* = 0.5, *Ec* = 0.0 and *Pr* = 2.

**Figure 7:**
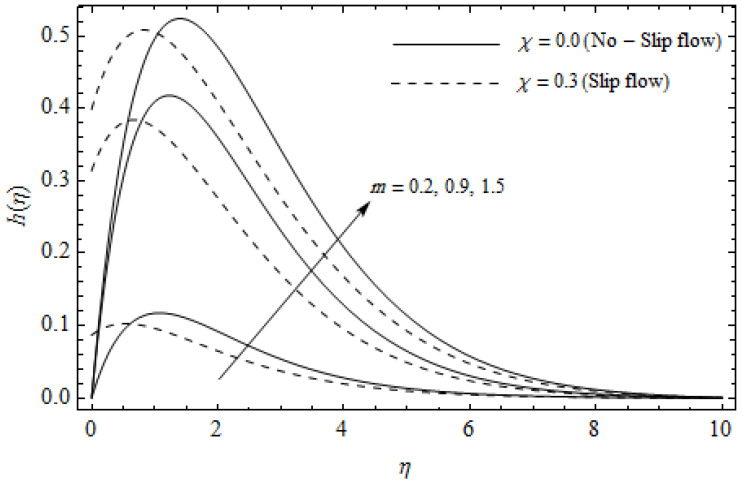
Secondary velocity profiles for different values of Hall parameter *m* and slip parameter χ with γ = 0.3, M=3, *m* = 0.5, *Ec* = 0.0 and *Pr* = 2.

**Figure 8:**
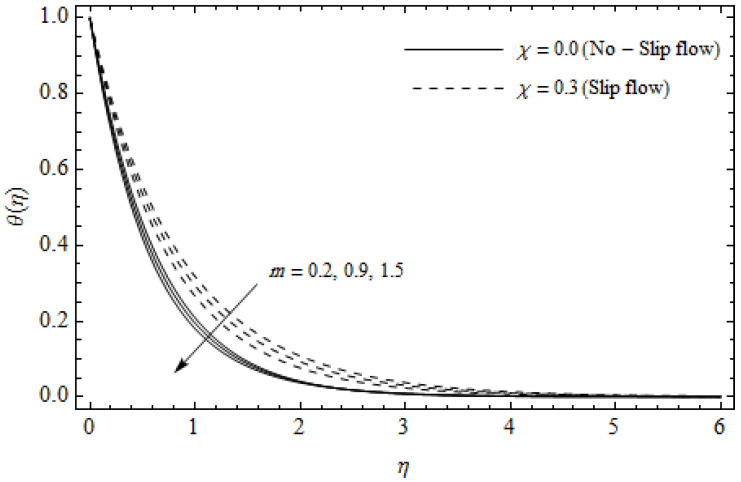
Temperature profiles for different values of Hall parameter *m* and slip parameter χ with γ = 0.3, M=3, *m* = 0.5, *Ec* = 0.0 and *Pr* = 2.

**Figure 9:**
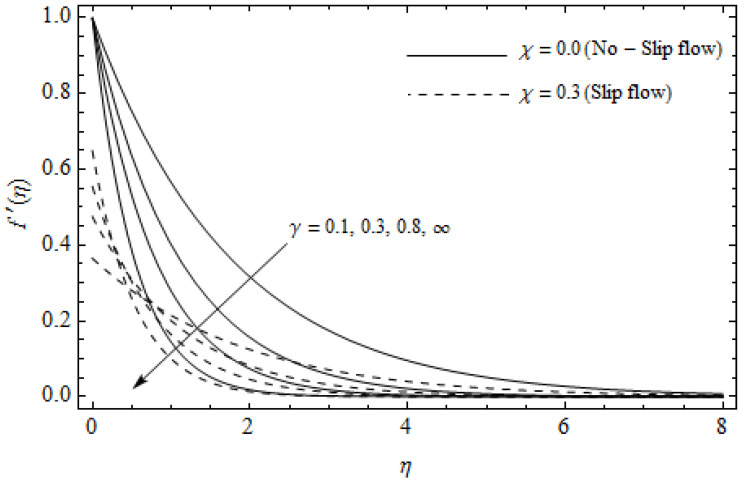
Axial velocity profiles for different values of Casson parameter γ and slip parameter χ with M=3, *m* = 0.5, *Ec* = 0.0, and *Pr* = 2.

**Figure 10:**
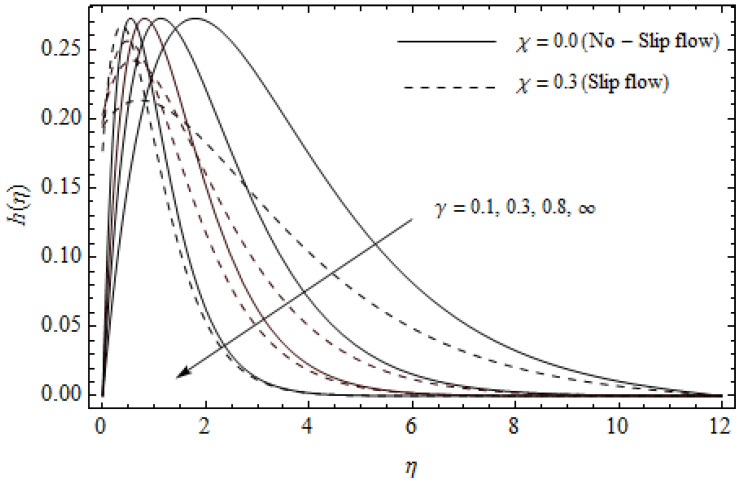
Secondary velocity profiles for different values of Casson parameter γ and slip parameter χ with *M* = 3, *m* = 0.5, *Ec* = 0.0 and *Pr* = 2.

**Figure 11:**
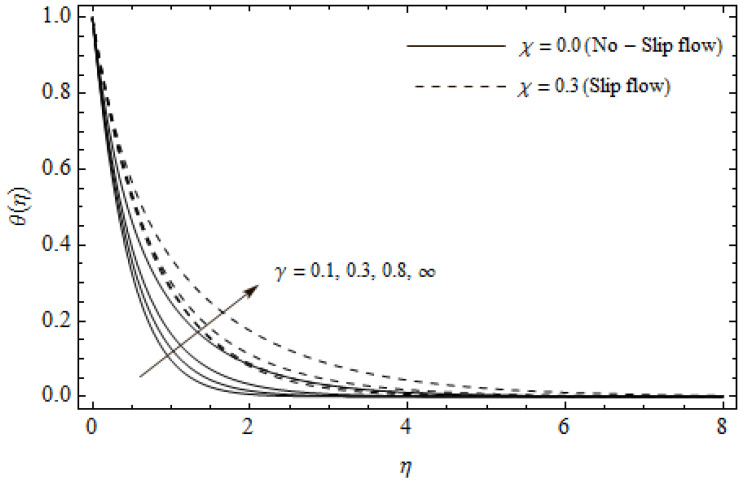
Temperature profiles for different values of Casson parameter γ and slip parameter χ with *M* = 3, *m* = 0.5, *Ec* = 0.0 and *Pr* = 2.

**Figure 12:**
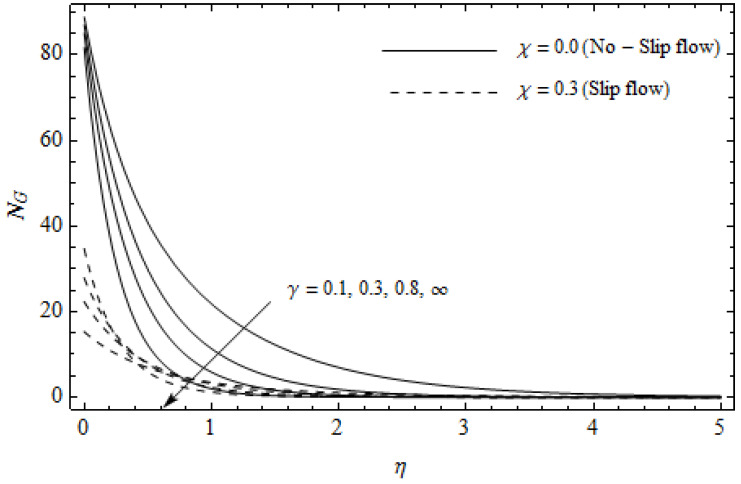
Effect of Casson parameter γ and slip parameter χ on NG with *M* = 3, *m* = 0.5, *Ec* = 0.2, *Pr* = 2, ReL=5 and $Br\Omega^{-1}=1$.

**Figure 13:**
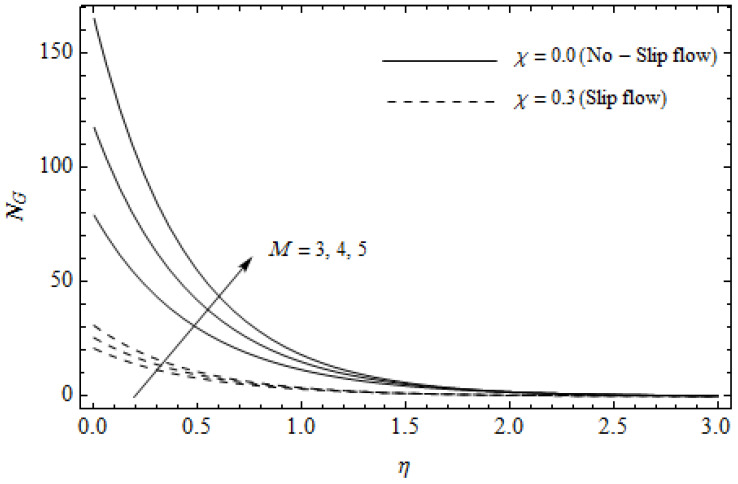
Effect of magnetic parameter *M* and slip parameter χ on entropy generation NG with γ = 0.3, *m* = 0.5, *Ec* = 0.2 and *Pr* = 2, ReL=5 and $Br\Omega^{-1}=1$.

**Figure 14:**
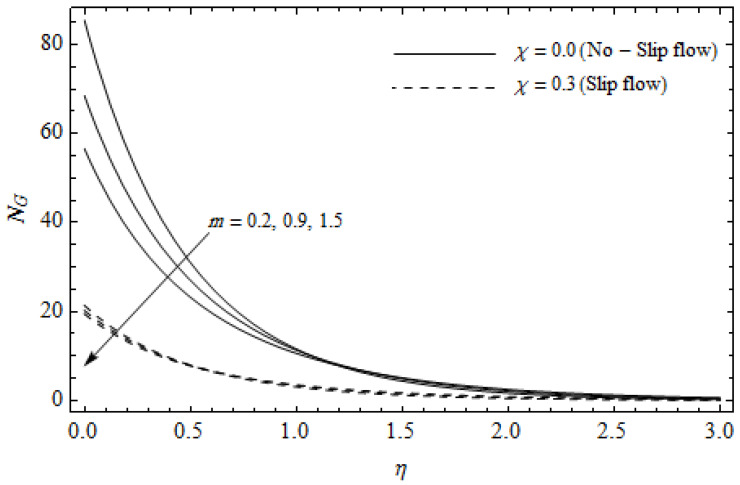
Effect of Hall parameter *m* and slip parameter χ on entropy generation NG with γ = 0.3, *M* = 3, *Ec* = 0.2, *Pr* = 2, ReL=5 and $Br\Omega^{-1}=1$.

**Figure 15:**
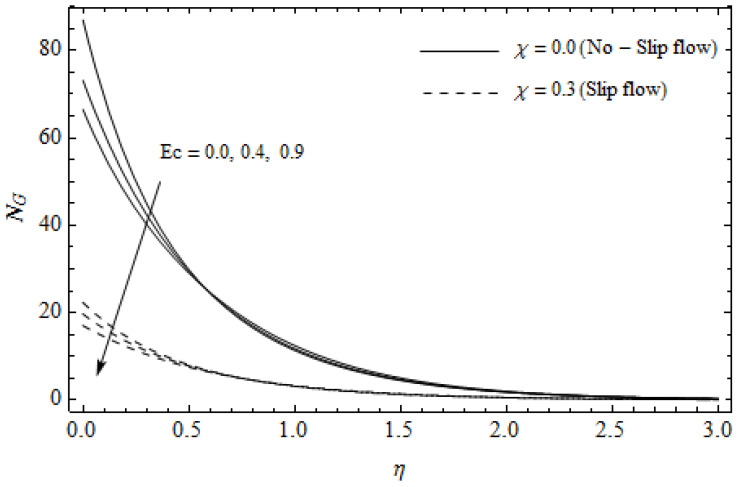
Effect of Eckert number Ec and slip parameter χ on entropy generation NG with γ = 0.3, M=3, m=0.5, *Pr* = 2, ReL=5 and $Br\Omega^{-1}=1$.

**Figure 16:**
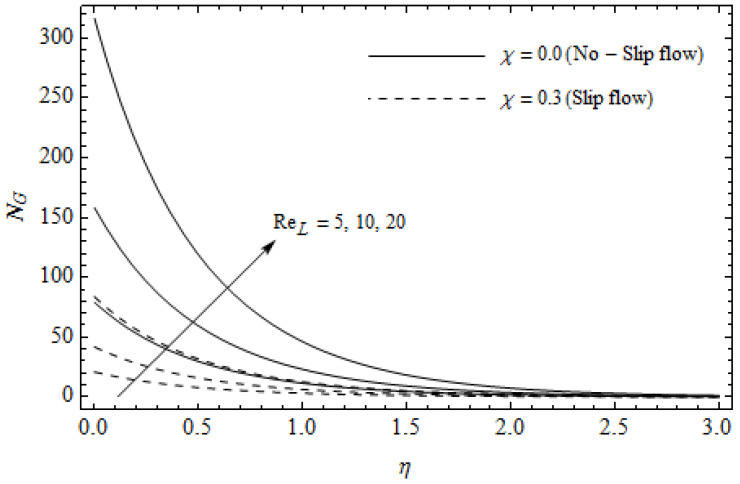
Effect of Reynolds number ReL and slip parameter χ on entropy generation NG with γ = 0.3, M=3, m=0.5, *Pr* = 2 and $Br\Omega^{-1}=1$.

**Figure 17:**
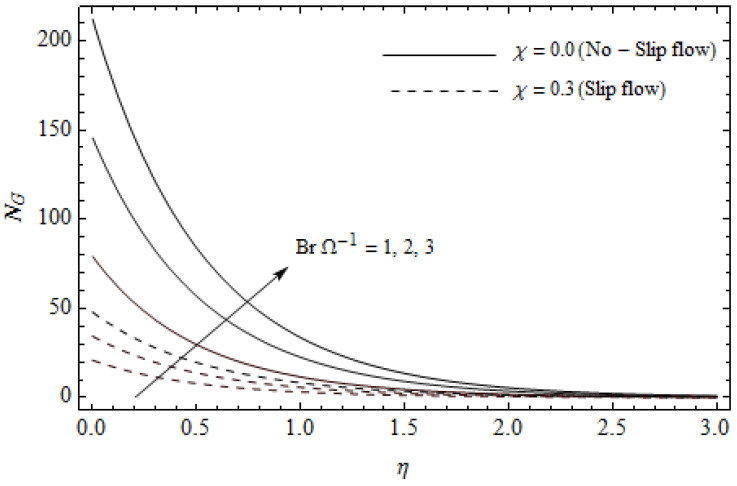
Effect of group parameter $Br\Omega^{-1}$ and slip parameter χ on the entropy generation NG with γ = 0.3, M=3, m=0.5, *Pr* = 2, and ReL=5.

**Figure 18:**
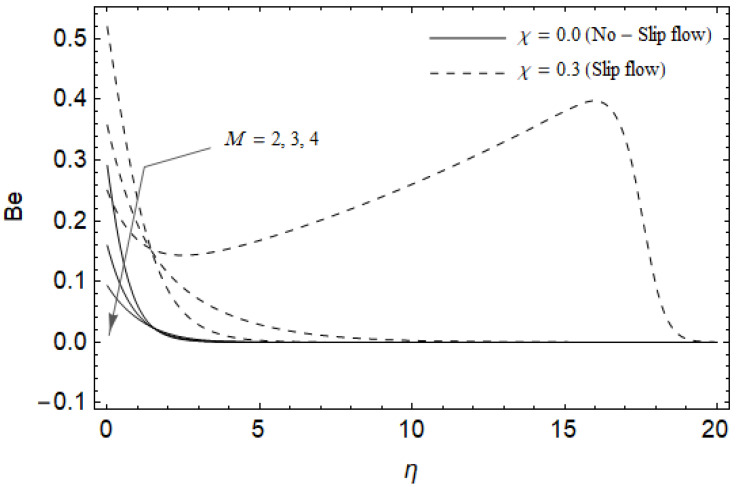
Effect of magnetic parameter M and slip factor χ on Bejan number *Be* with m=0.5, *Ec* = 0.0, γ=0.3, *Pr* = 2, ReL=5 and $Br\Omega^{-1}=1$.

**Figure 19:**
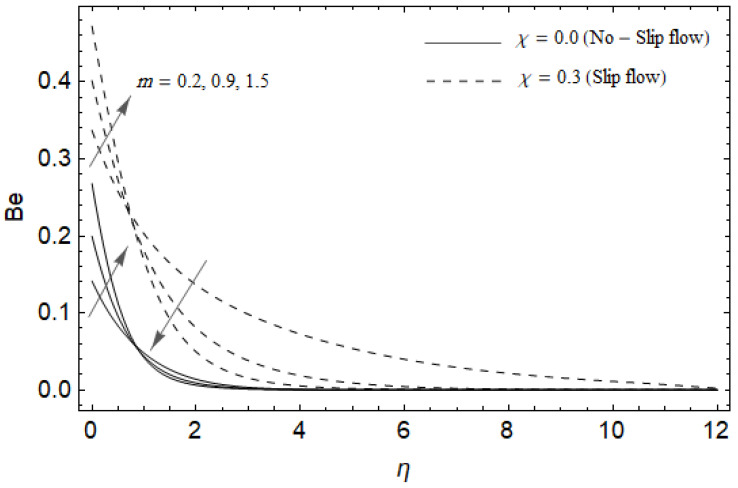
Effect of Hall parameter *m* and slip factor χ on Bejan number *Be* with *M* = 3, *Ec* = 0.0, γ=0.3, *Pr* = 2, ReL=5 and $Br\Omega^{-1}=1$.

**Figure 20:**
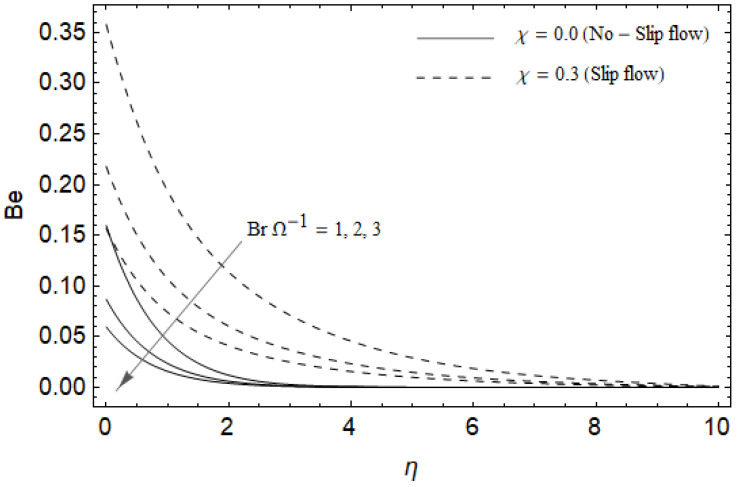
Effect of group parameter $Br\Omega^{-1}$ and slip factor χ on Bejan number *Be* with *M* = 3, *Ec* = 0.0, γ=0.3, *Pr* = 2, ReL=5 and m=0.5.

**Figure 21:**
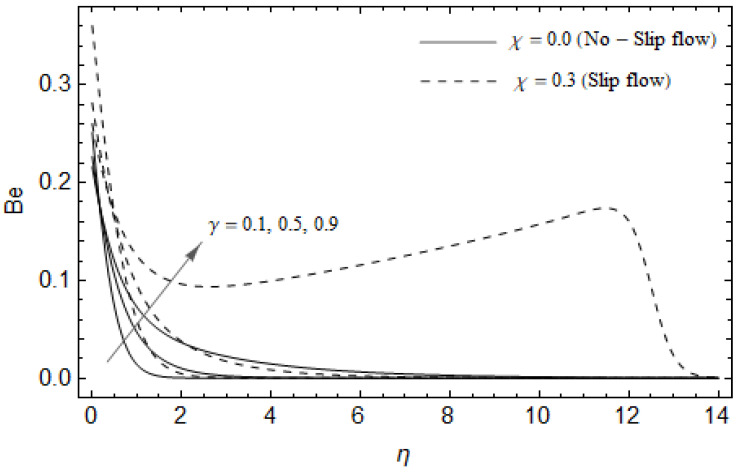
Effect of Casson parameter γ and slip factor χ on Bejan number *Be* with *M* = 3, *Ec* = 0.0, γ=0.3, *Pr* = 2, $Br\Omega^{-1}=1$, ReL=5 and m=0.5.

The emerging differential Equations (11)–(13) along with the relevant boundary conditions (14) are tackled numerically using the Runge-Kutta fourth order procedure with shooting technique. Numerical calculations were performed in the ranges 0.3≤γ≤∞, 3≤M≤5, 0.0≤m<1.5, 0.0≤χ≤0.7, 0.0≤Ec≤1.2, 5≤ReL≤20, $1\leq Br\Omega^{-1}\leq 3$ and Pr=2. Figure 2, Figure 3, Figure 4, Figure 5, Figure 6, Figure 7, Figure 8, Figure 9, Figure 10, Figure 11, Figure 12, Figure 13, Figure 14, Figure 15, Figure 16, Figure 17, Figure 18, Figure 19, Figure 20 and Figure 21 are plotted in order to see the impact of the magnetic parameter M, Hall parameter m, Eckert number Ec, Reynolds number ReL, group parameter $Br\Omega^{-1}$, and Casson parameter γ, respectively, on the primary velocity $f^{\prime }(\eta )$, secondary velocity h(η), temperature distribution θ(η), and entropy generation distribution NG as well as Bejan number Be. Further, the graphical results are presented in both cases of no-slip (χ=0) and slip boundary (χ≠0). 

### 4.1. Velocity and Temperature Profiles

Figure 2, Figure 3, Figure 4, Figure 5, Figure 6, Figure 7, Figure 8, Figure 9, Figure 10 and Figure 11 elucidate the influence of pertinent parameters on the velocity and temperature distributions. Figure 2, Figure 3 and Figure 4 show the effect of the magnetic parameter M for both cases of no-slip (χ=0) and slip boundary (χ≠0) on the primary velocity $f^{\prime }(\eta )$, the secondary velocity h(η), and the temperature profile θ(η). From Figure 2 and Figure 4, the primary velocity $f^{\prime }(\eta )$ reduces with an increase in M, whereas the reverse trend is seen for θ(η) in both cases. From Figure 3, the secondary velocity h(η) augments for larger values of M near the stretching sheet whereas it decays with an increase of η. This is attributable to the fact that the resistive Lorentz force owing to the magnetic field declines the fluid motion. This force helps to encourage the temperature profile. Both the velocity components within the boundary layers reduce with an increase in χ. On the contrary, increasing χ enhances θ(η) within the thermal boundary layer. Physically, the coupled effect of the slip factor and the magnetic field generate a retarding force. This retarding force allows more fluid to slip past the surface which decelerates the flow motion. Also, the temperature profile augments due to the occurrence of the force. The influence of the Eckert number Ec and slip parameter χ on the temperature field is displayed in Figure 5. Eckert number represents the kinetic energy of the flow relative to the boundary layer enthalpy difference. Enhancing Ec leads to a boost in thermal energy, which in turn elevates the temperature field for both cases. The thermal boundary layer thickness for the case (χ=0) is more pronounced than the case (χ≠0). Figure 6, Figure 7 and Figure 8 show the impact of Hall parameter *m* on the velocity components and the temperature field for two different cases. From Figure 6 and Figure 7, the velocity components enhance with an increase in *m* for both the cases. Physically, decreasing the conductivity $\left(\frac{\sigma }{1+m^{2}}\right)$ for rising values of *m* generates a magnetic damping force which boosts the velocity components of the fluid. It is also revealed that the velocity components are greater in the case (χ=0) in comparison to the case (χ≠0). It is noticed from Figure 8, that the temperature field θ(η) reduces with an increase in *m*. For the no-slip boundary case (χ=0), the temperature field is lower when compared to the case of slip boundary (χ≠0). The influences of Casson parameter γ on the primary velocity $f^{\prime }(\eta )$, the secondary velocity h(η) and the temperature distribution θ(η) for both cases (χ≠0) and (χ=0) are depicted in Figure 9, Figure 10 and Figure 11. Further, as γ→∞ the present problem reduces to the Newtonian fluid. From the figures, it is evident that the velocity components reduce with an increase in the parameter γ. Conversely, the temperature distribution is a growing function of the Casson fluid parameter γ, for both cases. This is owing to the fact that enhancing the values of γ augments the plastic dynamic viscosity and as a result, the yield stress dwindles. This creates resistance to the fluid motion and enhances the temperature distribution. It is interesting to see that increasing values of χ depresses both components of velocity whereas the opposite trend is observed for the temperature distribution. 

### 4.2. Entropy Generation (NG)

Figure 12, Figure 13, Figure 14, Figure 15, Figure 16 and Figure 17 portray the impact of pertinent parameters on the entropy generation. The influence of the Casson parameter γ and slip factor χ on the entropy generation NG is depicted in Figure 12. It is evident that an elevation in Casson parameter and slip factor diminishes the entropy generation gradually. We noticed that NG in the case of no-slip (χ=0) is greater than for the slip case (χ≠0). Figure 13 exhibits the variation of entropy generation NG with the magnetic parameter M and slip factor χ. From Figure 13, the entropy generation NG is an enhancing function of M whereas it is a decreasing function of χ. Physically, an increase of M generates a Lorentz force which increases the entropy production rate. This phenomenon shows that the magnetic force is a key principle in the entropy generation. Furthermore, the entropy generation profile NG for the case (χ=0) is more than that for the case (χ≠0). The entropy generation reduces with an increase of χ which reveals the system is cooling down. In Figure 14, the variations in the entropy generation profile are depicted for various values of Hall parameter *m* and slip factor χ. It is seen that the increase of *m* and χ decreases the entropy generation at the sheet nearby. This is due to the fact that the Hall current has considerable effects on Lorentz force term and current density. Consequently, increasing *m* augments effective electric conductivity which in turn depreciates NG and θ(η) as depicted in Figure 8. Further, the case of (χ=0) shows more impact on the entropy generation NG compared with the case (χ≠0). Figure 15 portrays the influence of Eckert number Ec and slip parameter χ on the entropy generation NG. It is interesting to see that enhancing values of Ec reduce NG near the surface and then rise in the region far away from the surface. For the case (χ=0), the entropy generation NG is more pronounced with a rise in Ec than the case (χ≠0). Figure 16 illustrates the impacts of Reynolds number ReL and slip parameter χ on the entropy generation NG. It reveals that NG is an increasing function of ReL. On the contrary, increasing values of χ reduce NG. Physically, the Reynolds number is represented by the ratio of inertial forces and viscous forces. Higher values of Reynolds number show the dominance of inertial forces which causes an enhancement in the entropy production. Further, it is remarkable that NG is higher in case (χ=0) than that in the case (χ≠0). Figure 17 is displayed to show the impact of group parameter $Br\Omega^{-1}$ and slip parameter χ on the entropy generation NG. From this figure, it is detected that NG boosts with an increase in $Br\Omega^{-1}$. However, the scenario becomes different with an increase in χ within the boundary layer region. Physically, increasing $Br\Omega^{-1}$ promotes the viscous effects of the fluid, which causes the entropy generation to enhance. The group parameter has a vital role to maximize the energy which measures the ratio of viscous effects and thermal asymmetry. On the other hand, it is noticed that the entropy generation is minimized with rising slip factor χ. This is due to the fact that the friction between the stretching surface and the fluid dwindles with an increase in χ. 

### 4.3. Bejan Number (Be)

Figure 18, Figure 19, Figure 20 and Figure 21 delineate the variation of the Bejan number for pertinent parameters. Figure 18 is plotted to depict the variation of Bejan number *Be* against magnetic parameter M and slip factor χ. Figure 18 shows that *Be* reduces with increasing M near the stretching sheet, but increases after a certain distance *η* from the stretching sheet for both cases. Physically, an increase in M leads to the irreversibility influences attributable to the fluid friction and the magnetic field becomes dominant in the neighborhood of the surface. For slip case (χ≠0), the Bejan number *Be* is more pronounced with the rise in M than the no-slip case (χ=0). The variations of *Be* with various values of the Hall parameter m and slip factor χ are plotted in Figure 19. From Figure 19, the Bejan number Be enhances with rising values of m near the stretching surface whereas the opposite trend occurs after a certain distance *η* from the boundary for both cases. It is observed that increasing m shows more impact on the Bejan number Be of the case (χ≠0) compared with the case (χ=0). Figure 20 is portrayed of Be against the group parameter $Br\Omega^{-1}$ and slip factor χ. From Figure 20, the Bejan number Be is a decreasing function of $Br\Omega^{-1}$ for both cases slip and no-slip. Physically, the rise in values of $Br\Omega^{-1}$ leads to promoting the fluid friction and magnetic field near the stretching surface which causes a reduction in Be. On the contrary, the heat transfer irreversibility is negligible (Refer Equation (22)). Notably, the Bejan number *Be* is more significant in the sense of magnitude for the slip case as compared to the no-slip case (χ=0) with increasing $Br\Omega^{-1}$. Figure 21 is plotted to show the variation of Be for various values of the Casson parameter γ and slip factor χ. From Figure 21, the Bejan number Be diminishes with an increase in γ near the stretching surface whereas the reverse behavior occurs after a certain distance *η* from the boundary for both cases of slip and no-slip. For the case (χ≠0), the Bejan number Be is enhanced with the rise in γ whereas the opposite trend is noticed for the case (χ=0).

### 4.4. Tables Discussion

Table 1 and Table 2, are constructed to display the numerical values of skin-friction $f^{\prime \prime }(0)$ and $h^{\prime }(0)$, as well as the heat transfer coefficient $\theta^{\prime }(0)$ for various values of the magnetic parameter M, Hall parameter m, Eckert number Ec and slip factor χ for both cases of Newtonian (γ→∞) and Casson flows. It is noticed that the magnitude values $f^{\prime \prime }(0)$ and $h^{\prime }(0)$ enhance gradually for rising values of M whereas the reverse scenario is noticed for $\theta^{\prime }(0)$ in both cases. Physically, increasing the magnetic parameter generates an electromagnetic force which depreciates the heat transfer rate while it augments both the magnitude values of the friction factor within the boundary layer. The values of $h^{\prime }(0)$ and $\theta^{\prime }(0)$ augment with increasing m whereas the reverse scenario is noticed for the magnitude values of $f^{\prime \prime }(0)$ for both cases. This is due to the fact that the electrical conductivity of the fluid declines with rising *m* which eventually dwindles the magnetic damping force. This serves to boost $h^{\prime }(0)$ and $\theta^{\prime }(0)$, but on the contrary, the magnitude values of $f^{\prime \prime }(0)$ reduce. From Table 1 and Table 2, the values of $\theta^{\prime }(0)$ boost whereas the coefficients $f^{\prime \prime }(0)$ and $h^{\prime }(0)$ are insensitive for rising Ec for both cases. Physically, the higher values of Eckert number *Ec* retard the fluid flow adjacent to the stretching surface. This agrees with the fact that the temperature distributions are enhanced with an increase in *Ec* as shown in Figure 5. Both the magnitude values of the friction factor $f^{\prime \prime }(0)$ and $h^{\prime }(0)$ as well as the heat transfer rate $\theta^{\prime }(0)$ reduce with increasing χ. Physically, with an increase in χ generates a resistive force neighboring to a surface which declines the physical quantities $f^{\prime \prime }(0)$, $h^{\prime }(0)$ and $\theta^{\prime }(0)$. For Casson flow, the values of skin-friction $f^{\prime \prime }(0)$ and $h^{\prime }(0)$ and the heat transfer coefficient $\theta^{\prime }(0)$ are more pronounced for all the previous physical parameters than Newtonian flow.

## 5. Conclusions 

In the present work, a numerical study of entropy generation on MHD Casson fluid with Hall current and slip factor has been addressed. The skin-frictions $f^{\prime \prime }(0)$, $h^{\prime }(0)$ and heat transfer coefficient $\theta^{\prime }(0)$, Bejan number Be and entropy generation NG are analyzed and represented through tables and graphs for various pertinent parameters. The significant outcomes are listed below:

1- The primary velocity $f^{\prime }(\eta )$ reduces with the rising of M, whereas the opposite behavior is observed for the temperature field θ(η).
2- The secondary velocity h(η) elevates with the rising of M near the stretching sheet whereas the reverse behavior occurs far away from the surface.
3- Both the velocity components $f^{\prime }(\eta )$ and h(η) enhance with an increase in *m* whereas the opposite scenario is observed for the temperature field θ(η).
4- Enhancing the values of Ec leads to boosting the temperature field θ(η).
5- Both the velocity components $f^{\prime }(\eta )$ and h(η) depreciate with an increase in γ whereas the reverse behavior is noticed for the temperature field θ(η).
6- Entropy generation NG augments for rising values of M, ReL, and $Br\Omega^{-1}$ whereas an opposite trend is remarkable for χ.
7- Entropy generation NG depreciates with increasing values of m, γ, Ec, and χ.
8- Bejan number *Be* reduces with rising M but increases after a certain distance *η* from the stretching sheet.
9- Bejan number *Be* enhances with rising m but depresses after a certain distance *η* from the stretching sheet.
10- Bejan number *Be* is a decreasing function of $Br\Omega^{-1}$.
11- Bejan number *Be* diminishes with a rise in γ near the stretching surface whereas the reverse behavior occurs after a certain distance *η* from the stretching sheet.
12- The impact of M, m, Ec and χ on the values $f^{\prime \prime }(0)$, $h^{\prime }(0)$ and $\theta^{\prime }(0)$ are more pronounced for Casson fluid when compared to the Newtonian fluid.
13- The magnitude values $f^{\prime \prime }(0)$ and $h^{\prime }(0)$ augment, whereas the values of $\theta^{\prime }(0)$ decrease with an increase in M.
14- The values of $h^{\prime }(0)$ and $\theta^{\prime }(0)$ enhance whereas the magnitude values of $f^{\prime \prime }(0)$ depreciate with increasing m.
15- The values of $\theta^{\prime }(0)$ enhance for large values of Ec.
16- Both the magnitude values of $f^{\prime \prime }(0)$ and $h^{\prime }(0)$ as well as $\theta^{\prime }(0)$ diminish with rising χ.

## Figures and Tables

**Table 1:** Variation of Rex Cfx, Rex Cfz and Rex−1/2 Nux for different values of *M*, *m*, *Ec*, χ and Pr=2.

(CassonFluid) γ=0.3						
*M*	*m*	*Ec*	χ	Rex Cfx	Rex Cfz	Rex−1/2 Nux
**3.0**	0.5	0.2	0.1	−2.74748	0.380355	1.45858
**4.0**	0.5	0.2	0.1	−2.98256	0.420435	1.36772
**5.0**	0.5	0.2	0.1	−3.18327	0.449734	1.28796
3.0	**0.2**	0.2	0.1	−2.84235	0.167365	1.43078
3.0	**0.9**	0.2	0.1	−2.56821	0.558594	1.51369
3.0	**1.5**	0.2	0.1	−2.31976	0.654144	1.59518
3.0	0.5	**0.0**	0.1	−2.74748	0.380355	1.69467
3.0	0.5	**0.6**	0.1	−2.74748	0.380355	0.98639
3.0	0.5	**1.2**	0.1	−2.74748	0.380355	0.27812
3.0	0.5	0.2	**0.0**	−3.90412	0.700737	1.58973
3.0	0.5	0.2	**0.4**	−1.48494	0.125011	1.13318
3.0	0.5	0.2	**0.7**	−1.02344	0.062368	0.93521

**Table 2:** Variation of Rex Cfx, Rex Cfz and Rex−1/2 Nux for different values of *M*, *m*, *Ec*, χ and Pr=2.

(Newtonian Fluid) γ→∞						
*M*	*m*	*Ec*	χ	Rex Cfx	Rex Cfz	Rex−1/2 Nux
**3.0**	0.5	0.2	0.1	−1.55680	0.244065	1.35999
**4.0**	0.5	0.2	0.1	−1.70926	0.278256	1.25131
**5.0**	0.5	0.2	0.1	−1.84307	0.305619	1.15369
3.0	**0.2**	0.2	0.1	−1.62314	0.108807	1.32919
3.0	**0.9**	0.2	0.1	−1.43584	0.349753	1.42218
3.0	**1.5**	0.2	0.1	−1.27717	0.395964	1.51636
2.0	0.5	**0.0**	0.1	−1.55680	0.244065	1.56711
2.0	0.5	**0.6**	0.1	−1.55680	0.244065	0.94577
2.0	0.5	**1.2**	0.1	−1.55680	0.244065	0.32444
2.0	0.5	0.2	**0.0**	−1.87548	0.336623	1.45527
2.0	0.5	0.2	**0.4**	−1.04359	0.120217	1.12444
2.0	0.5	0.2	**0.7**	−0.78951	0.072391	0.95809

## Nomenclature

B_0_	constant magnetic field (kg/s^2^ A)
b, c	positive constant
$c_{p}$	specific heat (J/kg K)
$C_{fx}$, $C_{fz}$	skin friction coefficients
Ec	Eckert number
$f^{\prime }(\eta )$	primary velocity
h(η)	secondary velocity
k	thermal conductivity of the fluid (W m^−1^ K^−1^)
M	magnetic parameter
m	Hall parameter
$Nu_{x}$	local Nusselt number
$p_{y}$	yield stress of the fluid
Pr	Prandtl number
ReL	Reynolds number
T	fluid temperature (K)
$T_{w}$	the temperature at the stretching surface (K)
$T_{\infty }$	the temperature at the stretching surface (K)
u,v,w	velocity components along x-, y-, z-axes (m s^−1^)
x,y,z	Cartesian coordinate (m)

## Greek Symbols

α	thermal diffusivity of the base fluid (m^2^ s^−1^)
γ	Casson parameter
χ	slip parameter
$Br\Omega^{-1}$	group parameter
η	similarity independent variable
θ	dimensionless temperature
μ	dynamic viscosity (kg m^−1^ s^−1^)
$\mu_{B}$	plastic dynamic viscosity of the non-Newtonian fluid
ρ	fluid density (kg m^−3^)
υ	kinematic viscosity (m^2^ s^−1^)
σ	the electrical conductivity of the fluid (s/m)

## Subscripts

w	quantities at the wall
∞	quantities far away from the surface
